# Cuticular differences associated with aridity acclimation in African malaria vectors carrying alternative arrangements of inversion 2La

**DOI:** 10.1186/1756-3305-7-176

**Published:** 2014-04-10

**Authors:** Kyanne R Reidenbach, Changde Cheng, Fang Liu, Cheng Liu, Nora J Besansky, Zainulabeuddin Syed

**Affiliations:** 1 Eck Institute for Global Health & Department of Biological Sciences, University of Notre Dame, Notre Dame, IN 46556, USA; 2 Department of Applied and Computational Mathematics and Statistics, University of Notre Dame, Notre Dame, IN 46556, USA; 3 Center for Research Computing, University of Notre Dame, Notre Dame, IN 46556, USA

**Keywords:** *An. gambiae*, *An. coluzzii*, Cuticular hydrocarbons, Chromosomal inversion, Cuticle, Desiccation resistance, GC-MS, M and S molecular forms

## Abstract

**Background:**

Principal malaria vectors in Africa, *An. gambiae* and *An. coluzzii*, share an inversion polymorphism on the left arm of chromosome 2 (2La/2L+^a^) that is distributed non-randomly in the environment. Genomic sequencing studies support the role of strong natural selection in maintaining steep clines in 2La inversion frequency along environmental gradients of aridity, and physiological studies have directly implicated 2La in heat and desiccation tolerance, but the precise genetic basis and the underlying behavioral and physiological mechanisms remain unknown. As the insect cuticle is the primary barrier to water loss, differences in cuticle thickness and/or epicuticular waterproofing associated with alternative 2La arrangements might help explain differences in desiccation resistance.

**Methods:**

To test that hypothesis, two subcolonies of both *An. gambiae* and *An. coluzzii* were established that were fixed for alternative 2La arrangements (2La or 2L+^a^) on an otherwise homosequential and shared genetic background. Adult mosquitoes reared under controlled environmental conditions (benign or arid) for eight days post-eclosion were collected and analyzed. Measurements of cuticle thickness were made based on scanning electron microscopy, and cuticular hydrocarbon (CHC) composition was evaluated by gas chromatography–mass spectrometry.

**Results:**

After removing the allometric effects of body weight, differences in mean cuticle thickness were found between alternative 2La karyotypes, but not between alternative environments. Moreover, the thicker cuticle of the *An. coluzzii* 2La karyotype was contrary to the known higher rate of water loss of this karyotype relative to 2L+^a^. On the other hand, quantitative differences in individual CHCs and overall CHC profiles between alternative karyotypes and environmental conditions were consistent with expectation based on previous physiological studies.

**Conclusions:**

Our results suggest that alternative arrangements of the 2La inversion are associated with differences in cuticle thickness and CHC composition, but that only CHC composition appears to be relevant for desiccation resistance. Differences in the CHC composition were consistent with previous findings of a lower rate of water loss for the 2L+^a^ karyotype at eight days post-eclosion, suggesting that CHC composition is an important strategy for maintaining water balance in this genetic background, but not for 2La. Despite a higher rate of water loss at eight days, higher body water content of the 2La karyotype confers a level of desiccation resistance equivalent to that of the 2L+^a^ karyotype.

## Background

A chromosomal inversion occurs when the chromosome breaks in two places and the intervening segment rotates 180° before rejoining. This results in a reversal of gene order without any change in gene content, aside from genes that may have been lesioned by the breakpoints themselves. Nevertheless, chromosomal inversions may have important phenotypic consequences, not least due to their effect on recombination. When evidence for chromosomal inversions was first documented in 1921, the potential for reduced recombination in individuals heterozygous for alternative gene arrangements was immediately hypothesized [1]. It is now a tenet of classical genetics that a single crossover between inverted and uninverted arrangements within the inversion loop that forms during meiotic chromosomal pairing results in genetically unbalanced and inviable recombinant gametes, which are thus not recovered. It is this characteristic of effective suppression of recombination by inversions that has particularly interested evolutionary biologists, due to the potential for a chromosomal inversion event to capture between its breakpoints locally adapted alleles at multiple loci, and maintain their association against genetic exchange with deleterious immigrant alleles carried on an uninverted chromosome [2,3]. In this way, inversions may facilitate adaptive evolution in heterogeneous environments [2,4].

The notion that inversions are not neutral population genetic markers, but may be maintained by strong natural selection, arose from cytogenetic studies of *Drosophila* based on the banding pattern of giant larval salivary polytene chromosomes [5-7]. Species such as *Drosophila pseudoobscura*, *D. subobscura*, *D. robusta* and *D. melanogaster* are polymorphic for chromosomal inversions whose frequencies vary clinally along environmental gradients, cycle regularly with the seasons in concert with climatic variables [8], and shift in response to global warming [9,10], suggesting that the adaptive benefit of alternative arrangements is dependent on local environmental conditions. One of the most compelling examples is inversion In(3R)P in *D. melanogaster*, which shows parallel clinal patterns along the eastern coasts of two different continents, from temperate regions where the inversion is rare, to tropical regions where its frequency is very high [11,12]. Traits associated with inversion polymorphism in *Drosophila* include development time, body size, wing shape and thermal resistance, among others [3]. Inversion polymorphism in a variety of other insects has also been associated with many of the same traits [3]. Studies of inversion-trait associations in *Anopheles* mosquitoes have documented that wing shape in *Anopheles funestus*[13] and resistance to heat, desiccation, and insecticides in *An. gambiae* and *An. coluzzii*[14-17] are linked to inversion polymorphisms.

*Anopheles gambiae* and *An. coluzzii* (formerly *An. gambiae* S and M molecular forms; [18]) are principal vectors of human malaria in Africa, where 90% of the >600,000 deaths from malaria occur annually [19]. Abundant inversion polymorphism in these species likely contributes to their successful exploitation of vast areas of sub-Saharan Africa, spanning diverse ecoclimatic zones from mesic rainforest to xeric savanna, but always in close association with humans [20,21]*.* One of the oldest, most geographically widespread, and best-studied polymorphic inversion systems in these mosquitoes, 2La and its alternative 2L+^a^, is shared by common ancestry between *An. gambiae* and *An. coluzzii*[22]. Frequencies of 2La are correlated with degree of aridity. This has been documented in West and Central Africa along environmental gradients of aridity from the mesic rainforest belt flanking the Gulf of Guinea where 2La is rare or absent, to xeric sahel savanna regions hundreds of kilometers to the north where 2La reaches fixation [23,24]. Longitudinal studies in localities where 2La is polymorphic have demonstrated that its frequency fluctuates in a predictable pattern tied to annual rainfall variation between rainy and dry seasons [25,26]. Even at the scale of a village, 2La is non-randomly distributed among mosquitoes resting indoors versus outdoors at night, a pattern attributed to the higher saturation deficit found inside dwellings where 2La is overrepresented [27]. DNA sequencing studies of natural *An. gambiae* populations sampled along a cline of aridity in Cameroon support the notion that spatially varying selection is maintaining the 2La inversion polymorphism [23,28]. Among the candidate genic targets of selection in 2La are cuticle protein (CPR) genes, many of which occur in a large cluster near the distal breakpoint [23,28].

The strong correlation between 2La frequency and degree of aridity suggests that alleles within the 2La inversion may confer resistance to thermal and/or desiccation stress, both of which are environmental stressors routinely encountered by insects. Recent physiological studies have confirmed the association of 2La with thermal tolerance of *An. coluzzii* larvae [14] and desiccation resistance of both *An. coluzzii* and *An. gambiae* adults [15,17]. In particular, adult females homozygous for the 2La inversion were significantly more resistant to desiccation (measured as time to death in dry air), due to lower rates of water loss in newly emerged adults, and higher total body water content at four days post-emergence [17]. Interestingly, by eight days post-emergence, there was no significant difference in survival between the karyotypes under desiccation stress. At this time point, the higher initial water content of 2La karyotypes was balanced by their higher rate of water loss relative to 2L+^a^—a pattern confirmed in eight-day old females acclimated to a lower humidity regimen [17]. Prior acclimation to non-lethal desiccation stress improved desiccation resistance for females of both karyotypes, and although subsequent resistance to lethal desiccation stress was comparable between alternative karyotypes at eight days post-emergence, the orientation of the 2La rearrangement was associated with opposing effects on the storage of energy reserves in acclimated females. While 2La inverted homokaryotypes boosted their mass-specific glycogen content and reduced lipid stores, the converse was true for 2L+^a^ homokaryotypes [17]. It is noteworthy that increased storage of glycogen, utilized by *Drosophila* under desiccation stress [29], has been associated with higher dehydration tolerance in flies [29-31], consistent with its proposed role as an osmolyte for water retention [30]. Taken together, these data suggest that genes inside the 2La inversion influence water balance and desiccation resistance, but also that resistance to desiccation is a complex trait, whose precise physiological basis and causal genes remain to be elucidated.

One of the main barriers to water loss in insects is the cuticle [32-35], which may be modified to enhance desiccation resistance. Increases in the amount of surface lipids (mainly cuticular hydrocarbons, CHCs) or changes in their chemical composition resulting in increased chain length, linearity, and saturation are the main means of minimizing cuticular transpiration in insects [32,36]. Another non-mutually exclusive mechanism that may further reduce cuticular permeability is thickening of the cuticle. Increased cuticle thickness is associated with pyrethroid resistance in *An. funestus*[37], and structural cuticular proteins implicated in pyrethroid resistance in two other anophelines, *An. gambiae*[38] and *An. stephensi*[39], are found in the endocuticle where they may contribute to cuticle thickening [40]. In the Colorado potato beetle, transcripts encoding cuticular proteins were highly induced not only by insecticide treatment of resistant beetles but also by arid conditions, suggesting that increased cuticle thickness may be a common adaptive response to both environmental stresses [41].

A possible association of the 2La inversion system with cuticular characteristics of *An. gambiae* and *An. coluzzii* has not been investigated before now. The objective of this study was to assess karyotype-dependent effects on cuticle thickness as well as amount and composition of surface lipids in adults maintained under controlled environmental conditions.

## Methods

### Origin, husbandry and experimental treatments of mosquito colonies

Sub-lines of *An. gambiae* and *An. coluzzii* homokaryotypic for alternative arrangements of 2La (*i.e*., 2La/a and 2L+^a^/+^a^) were derived from parental colonies that were polymorphic for 2La. Apart from the alternative arrangements of 2La, the two sub-lines of each species that were employed for this study share the same genetic background. The *An. coluzzii* sub-lines SUCAM-2La and SUCAM-2L+^a^ were derived in 2008 from a parental colony (SUCAM) established in 2005, as described previously [17]. Using a similar approach, the homokaryotypic *An. gambiae* sub-lines NDKO-2La;2R+^b^ and NDKO-2L+^a^;2R+^b^ were derived from a parental colony (NDKO) established in 2008 from mosquitoes collected in Ndokayo, Cameroon [15]. From the parental NDKO colony carrying 2La/2L+^a^ and 2Rb/2R+^b^ inversion polymorphisms, four homokaryotypic sub-lines were derived by molecular karyotyping of virgin males and females, as follows. On the morning of adult emergence, virgin females and males were separated into individual vials. DNA extracted from one rear leg of each mosquito [42] was used for molecular karyotyping of 2La and 2Rb, performed in separate PCR reactions following White *et al.*[43] and Lobo *et al.*[44]. The second day, successfully karyotyped males and females were sorted into four population cages according to karyotype and allowed to mate: (1) 2La/a;2Rb/b, (2) 2La/a;2R+^b^/+^b^, (3) 2 L+^a^/+^a^;2Rb/b, and (4) 2L+^a^/+^a^;2R+^b^/+^b^. Females were offered blood meals to support egg production, and the progeny of each population cage were reared to adults. A random subset (N = 20) of the adult female progeny from each of the four populations was karyotyped by polytene chromosome analysis [45] for each of three successive generations, to ensure the absence of inversion loops (indicative of heterokaryotypes). Experiments were started at least three generations after derivation of the NDKO-2La/2R+^b^ and NDKO-2L+^a^/2R+^b^ homokaryotypic sub-lines used in this study.

For routine colony husbandry, and for controlled experimental conditions referred to as “benign”, adult mosquitoes were held in insectaries maintained at 27°C and 85% relative humidity with a 12 h:12 h light:dark cycle and 1 h crepuscular (dawn and dusk) transitions. Non-lethal desiccation stress, referred to as “arid” experimental conditions, was imposed on adult females held in an insectary chamber maintained at 30°C and 60% RH. Immature stages of all sub-lines and both treatment groups were reared under common benign insectary conditions. Eggs were placed in plastic trays (27 × 16 × 6.5 cm) containing 2 L of water purified by reverse osmosis. Larvae were reared at equal density (200 per pan) and fed 180 mg of 2:1 finely ground tropical fish pellet:bakers yeast, every other day until day five, and daily thereafter until pupation. Pupae were transferred to 0.2 m^3^ emergence cages. Within 24 h of emergence, mosquitoes were sexed and virgin females were transferred to cages where they were held for eight days, either under “benign” or “arid” conditions. Adult females had free access to a 10% corn syrup solution supplied through saturated cotton balls provided fresh daily.

### Cuticle thickness and total body dry mass measurements

At eight days post-emergence, at least 14 adult females from eight treatment groups (2 species x 2 karyotypes x 2 environmental conditions) were cold euthanized. Both mesothoracic legs of freshly killed mosquitoes were removed and preserved in 80% ethanol until they could be processed for scanning electron microscopy (SEM). The remaining carcass was desiccated overnight in an oven set to 70°C, and subsequent dry weight was determined using a microbalance (±0.2 μg, Mettler-Toledo, Columbus, OH, USA). Cuticle thickness was estimated by SEM from a cut through the left mesothoracic leg, following Wood *et al*. [37]. First, the left leg was brushed gently with a small paintbrush to remove the scales. The first tarsal segment was cut at the midpoint with a new platinum coated razor blade. Both resulting leg fragments were mounted cut side up on carbon tape attached to an aluminum stub mount, and coated with 4 μm of iridium using a Cressington 208 HR sputter coater (Cressington Scientific Instruments, Watford, UK) in conjunction with the Cressington MTM 20 thickness monitor. Images of the cut of both leg fragments were taken with a FEI-Magellan 400 FESEM (FEI, Hillsboro, OR, USA), and the highest quality image chosen for measurement. If a high quality image of the left leg could not be obtained, the right leg was processed in the same manner.

**Figure 1:**
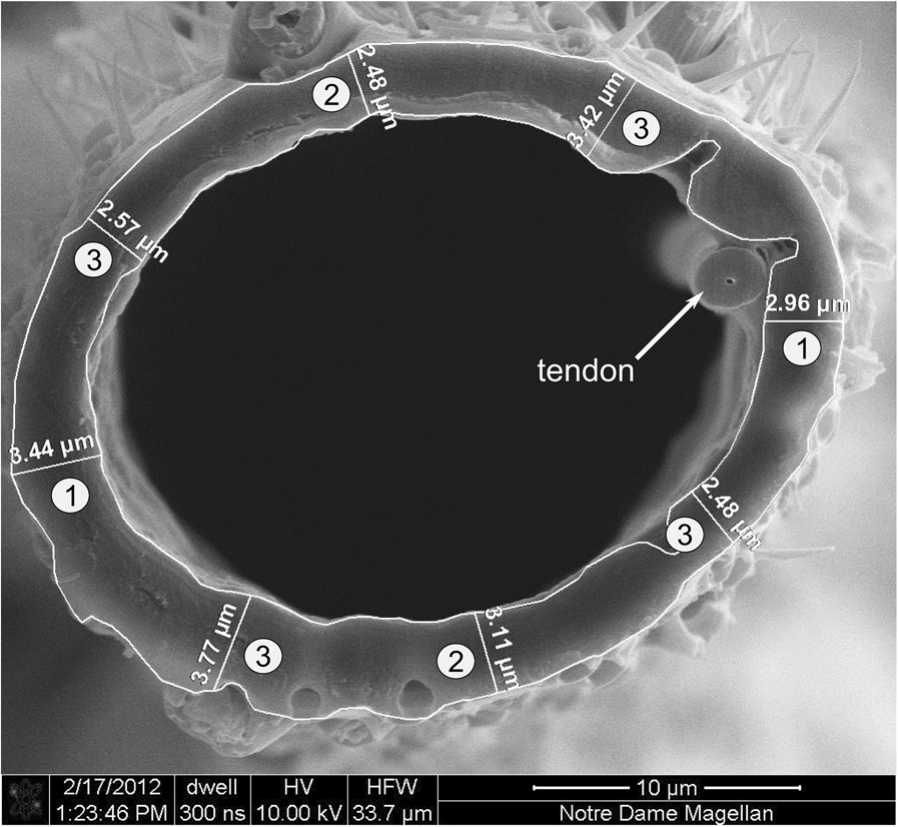
**Scanning electron micrograph showing a representative cross-sectional view of a mesothoracic leg used to estimate cuticle thickness.** Overlaid in white are the outlines of the inner and outer cuticle borders along with the initial eight measurements of cuticle thickness. Measurements were taken starting near the tendon and the section immediately across (1). The halves created by this division were subsequently divided four additional times. Only the first two divisions (2 and 3) are indicated here.

Digital micrographs were analyzed using Zeiss AxioVision software. First, the inner and outer cuticle boundaries were delineated. The first two cuticle thickness measurements consisted of the length between the inner and outer boundary near the tendon and its mirror on the opposite side. Two additional thickness measurements were taken at points along the circumference midway between the first two measurements, and this process was repeated three more times to yield a maximum of 32 measurements (Figure 1). Sections containing cuticle imperfections that would impair measurements were bypassed, and images yielding less than 16 reliable measurements were discarded. Cuticle thickness for each female mosquito was reported as the mean value of 16–32 thickness measurements around the cuticle circumference.

### Cuticular hydrocarbon (CHC) characteristics

Samples from a total of four treatments (2 karyotypes of *An. coluzzii* SUCAM x 2 environmental conditions) with five replicates each were analyzed on a coupled gas chromatography–mass spectrometer (GC-MS). Each replicate consisted of 10 virgin females. Total CHCs were extracted from a pool of 10 virgin females for each replicate by immersing the mosquitoes for 10 min in 200 μl of glass-distilled hexane (Fischer, ≥98.5% purity) in a 3 ml Wheaton® high recovery NextGen™ V-Vial attached with PTFE lined septa caps (Wheaton, Millville, NJ). Prior to their removal, mosquitoes were rinsed with an additional 150 μl of hexane. Hexane extracts were then evaporated under a gentle stream of charcoal filtered air using a Syntech Stimulus Controller CS-55 (Syntech, Kirchzarten, Germany). CHC extracts were reconstituted with 20 μl of hexane containing 10 ng/μl of octadecane as an internal standard (IS). Octadecane has widely been used as an IS in similar CHC analyses, and it was also chosen because its retention time did not overlap with any of the hydrocarbons in our biological samples.

Chemical analyses were performed on a 7890A GC system (Agilent Technologies, Santa Clara, CA) coupled with a 5975C Agilent Technologies mass spectrophotometer (inert XL MSD with a triple-Axis Detector). One microliter of an extract was injected in a split-splitless injector operating under splitless mode at 250°C in an Agilent HP-5 capillary column (30 m, 0.32 mm ID, 0.25 μm phase thickness). Helium (Ultra High Purity 5.0 Grade; Airgas, USA) was used as the carrier gas at a constant flow rate of 1 ml/min. The column was held isothermally at 70°C for 1 minute, then programmed to increase at a rate of 10°C per minute until 310°C, with a final hold of 10 minutes. MS was operated at 70 eV. Data recording and quantification was performed using Agilent MSD ChemStation software (E.02.02.1431). Initial chemical identity was determined using NIST 2011 MS library.

Synthetic hydrocarbons of known carbon chain lengths from C_10_ to C_34_ were run to determine Kovat’s Retention Index (RI). There were sixteen constituent peaks that consistently appeared in the chromatograms of each pool, and these were selected for further analyses since their amounts were well above the detection limit of the MS (as inferred from the IS amounts). Identification of these CHC was made in two ways: 1) comparison of the RI calculated for each of the 16 compounds relative to known RIs from Pherobase (http://www.pherobase.com); and 2) confirmation of retention times and their mass spectra compared with synthetic standards.

For each replicate, the area under each CHC peak was quantified and expressed as a proportional value relative to the sum of all peaks. Relative abundance values were Gaussian-transformed to normalize the data prior to analysis.

### Statistical analyses

Statistical analyses were conducted using R v2.13.1 and SAS 9.2 (SAS Institute, Cary, NC).

### Cuticle thickness

Initial exploration of the relationship between total body dry weight and cuticle thickness was based on the Pearson’s product moment correlation by species and environmental condition (four groups). An allometric relationship between body weight and cuticle thickness meant that variation in body weight among individuals in a group would confound inferences about the direct contribution of karyotype to cuticle thickness. Accordingly, the normalization approach described by Lleonart *et al.*[46] to remove allometric effects of body size was adapted for the general linear modeling framework applied here. Specifically, the initial general linear models, by species and condition, contained log-transformed cuticle thickness as the response variable, and log-transformed total dry body weight, karyotype, and their interaction as the independent variables. As the interaction terms from all four initial models were not statistically significant (all *P*-values from the t-tests of interaction were > 0.20), they were removed from the final models. From each of the four models, the least-squares means and 95% confidence intervals (CI) were obtained for log-transformed cuticle thickness by karyotype, which were then back-transformed to the linear scale (estimates known as geometric means) for reporting purposes. Statistical significance of the difference in standardized cuticle thickness between karyotypes for each species and condition (four comparisons) was determined from *P*-values based on t-statistics.

### Cuticular hydrocarbons

Statistical significance of differences in the amounts of individual compounds was assessed using ANOVA models that included karyotype, environmental condition, and their interaction as main (fixed) factors. Quantitative differences in overall CHC profiles were analyzed by principal component analysis (PCA). The first four PCs with eigenvalues greater than 1 explained 88% of the variance. Multivariate analysis of variance (MANOVA) with these first four PCs as response variables, and karyotype, environmental condition, and their interaction as factors was conducted to test for statistical significance of differences due to karyotype and environment.

## Results

Using the same SUCAM colony of *An. coluzzii*, Gray *et al.*[17] showed that at eight days post-emergence, females homokaryotypic for the 2La inversion lost water at a significantly higher rate (standardized for body mass) than their 2L+^a^ counterparts, despite an otherwise common genetic background. Although acclimation to arid environmental conditions reduced the rate of water loss relative to non-acclimated females regardless of karyotype, the difference between karyotypes—whereby female carriers of 2La lost water at a significantly higher rate—was consistent with or without acclimation. Accordingly, to assess whether changes in cuticle characteristics could be implicated in karyotype-based differences in standardized rates of water loss, we employed the same sex, age and experimental conditions as in Gray *et al.*[17].

### Positive allometry of cuticle thickness and total body dry weight

**Figure 2:**
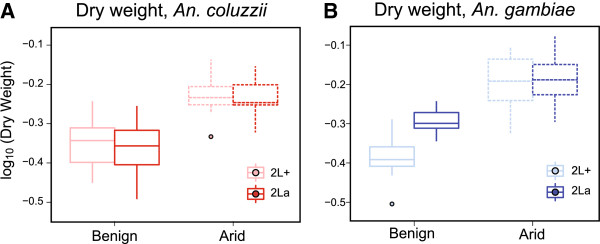
**Box-and-whisker plots of total body dry weight.** Plots illustrate the spread of log-transformed data from *An. coluzzii* SUCAM **(A)** and *An. gambiae* NDKO **(B)** adult females carrying alternative homokaryotypic arrangements of 2La, held for eight days under benign or arid conditions. The horizontal line indicates the median, box length represents the inter-quartile range (IQR), and whiskers span the range of data within +/- 1.5*IQR of the 1^st^ and 3^rd^ quartiles. Data outside of this range are represented as points.

**Figure 3:**
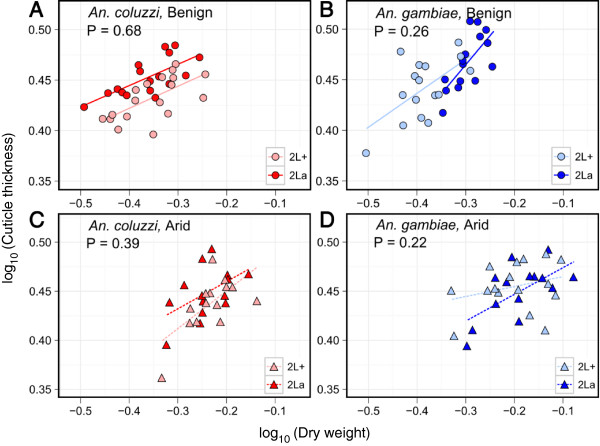
**Log-log relationships of cuticle thickness (μm) versus total body dry weight (mg) for two species under two environmental regimens.** Plots **(A, B)** illustrate *An. coluzzii* and *An. gambiae* under benign conditions; plots **(C, D)** illustrate these species under arid conditions. Each data point (circles and triangles for benign and arid conditions, respectively) represents an individual adult female. *P*-values in each plot indicate that the slopes of the regression lines do not differ significantly between alternative homokaryotypes (2La versus 2L+^a^, represented by dark versus light shaded lines and symbols) of the same species in the same environment.

Adult female mosquitoes maintained for eight days from the time of eclosion under benign or arid insectary conditions were evaluated in two species, *An. coluzzii* (SUCAM) and *An. gambiae* (NDKO), to assess whether the orientation of the 2La chromosomal rearrangement impacted cuticle thickness. Total body dry weight was recorded together with cuticle thickness for the 14–18 individual females included in each of eight treatment groups (2 species x 2 karyotypes x 2 environmental conditions), in anticipation that body size would co-vary with cuticle thickness. Mean measurements of cuticle thickness and total body dry weight for each of the eight groups are provided in Table 1. Assessed on its own, total body dry weight was significantly higher for females acclimated to arid conditions relative to those maintained under benign conditions, and this trend applied for both species regardless of karyotype (*P* < 0.0001 for each karyotype/species; Figure 2). Moreover, initial tests of the expected positive relationship between dry weight and cuticle thickness for adult females maintained under the same environmental condition revealed a significant correlation in both species (Table 2). This relationship is illustrated in Figure 3 by logarithmic scatter plots of cuticle thickness by total body dry mass for each of the eight treatment groups. The t-tests of interaction between log(dry weight) and karyotype for the four species and condition combinations yielded non-significant *P*-values in all cases (Figure 3A-D), indicating that for a given species and environmental condition, the slopes of the regression lines for alternative karyotypes were indistinguishable statistically.

**Table 1:** Sample size and means for cuticle thickness and dry weight for females in each of the eight treatment groups

**Species**	**Karyotype**	**Environment**	**N**	**Mean cuticle thickness (μm) ± SE**	**Mean dry weight (mg) ± SE**
*coluzzii*	2L+^a^	benign	18	2.71 ± 0.031	0.45 ± 0.015
	2La	benign	17	2.84 ± 0.030	0.44 ± 0.015
	2L+^a^	arid	15	2.75 ± 0.043	0.59 ± 0.016
	2La	arid	14	2.82 ± 0.045	0.58 ± 0.017
*gambiae*	2L+^a^	benign	16	2.77 ± 0.047	0.42 ± 0.013
	2La	benign	15	2.94 ± 0.047	0.51 ± 0.010
	2L+^a^	arid	16	2.85 ± 0.040	0.64 ± 0.025
	2La	arid	15	2.83 ± 0.045	0.66 ± 0.023

**Table 2:** Pearson’s correlation between cuticle thickness and total body dry weight for females from each species and environmental condition

**Species**	**Environment**	**r**	***P*-value**
*coluzzii*	benign	0.548	0.001
	arid	0.480	0.007
*gambiae*	benign	0.670	0.000
	arid	0.422	0.018

### Inversion-specific effects on cuticle thickness

**Figure 4:**
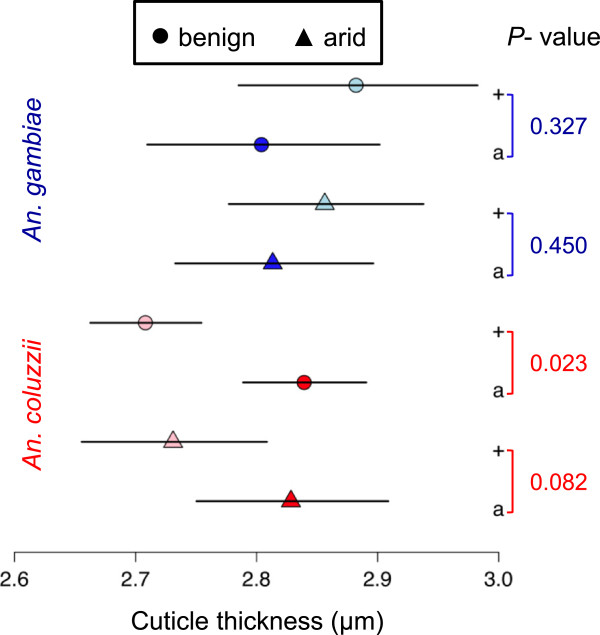
**Least-squares geometric mean differences in cuticle thickness standardized for weight.** Shown are least-squares means, symbolized by shapes and colors for condition and species, respectively: benign, circles; arid, triangles; *gambiae*, blue; *coluzzii*, red. Horizontal lines represent the 95% confidence interval. Statistical significance of the difference in cuticle thickness between alternative karyotypes for each species and condition was determined based on t-statistics (see Methods).

After removing the allometric effect of body size (see Methods), the resulting least-squares geometric means of cuticle thickness were compared between karyotypes, for each species and environmental condition (Figure 4). For *An. gambiae* NDKO, mean cuticle thickness between karyotypes was not statistically significant owing to strongly overlapping 95% confidence intervals. In contrast, for *An. coluzzii* SUCAM there was a statistically significant difference in cuticle thickness between 2L+^a^ and 2La under benign conditions, and a marginally non-significant difference (*P* = 0.08) between the karyotypes under arid conditions (Figure 4). In this case, mean cuticle thickness was greater for the 2La karyotype under both benign and arid conditions.

### Inversion-dependent effects on cuticular hydrocarbon composition

**Figure 5:**
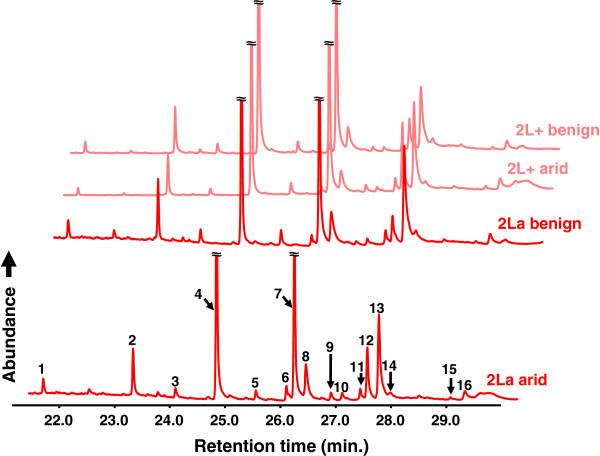
**Sample *An. coluzzii *cuticular hydrocarbon profiles representative of each karyotype and environmental condition.** Total ion chromatograms are offset and truncated for presentation purposes.

**Figure 6:**
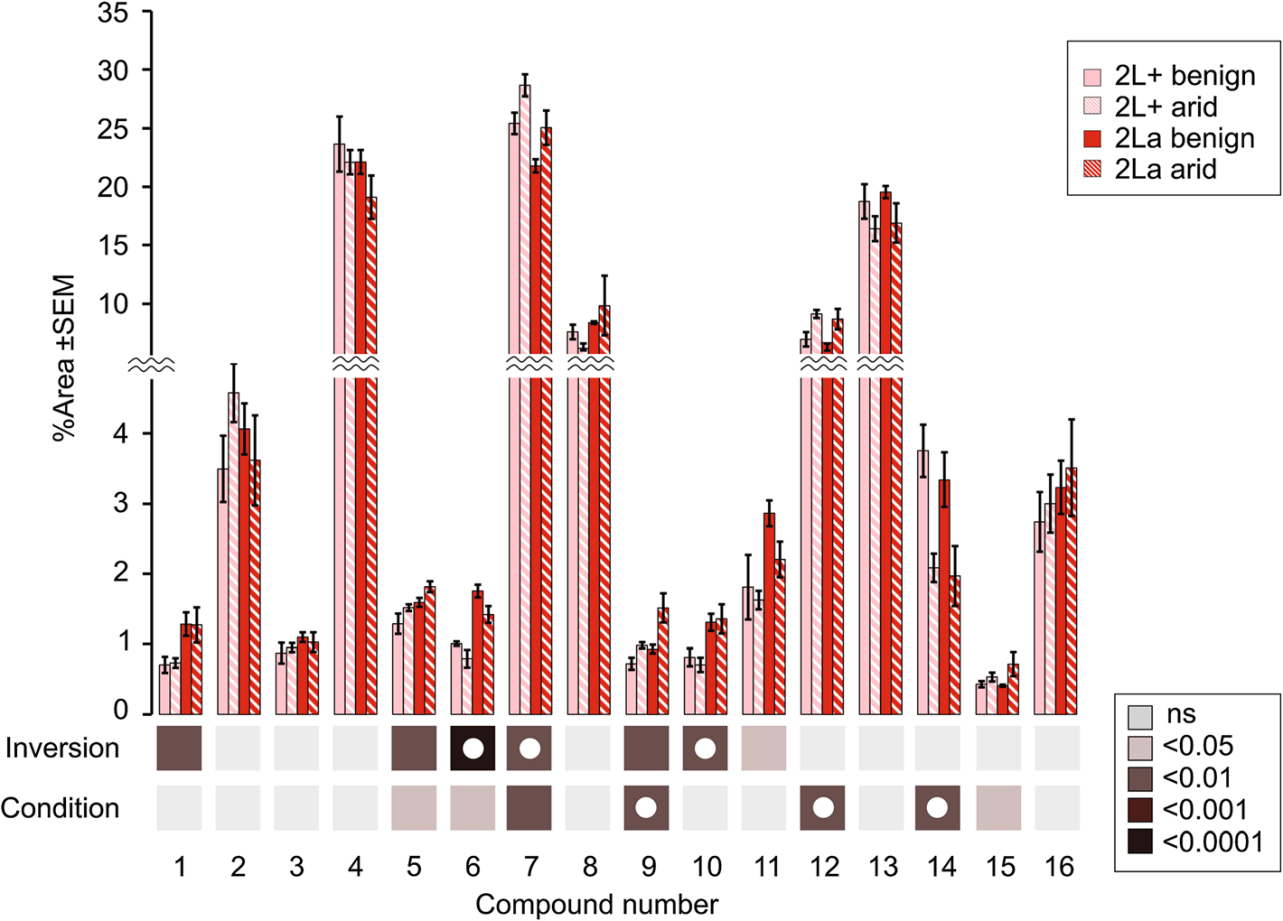
**Mean relative abundance (±SE) of the 16 cuticular hydrocarbons of *An. coluzzii *SUCAM detected for each karyotype and environmental condition.** Beneath the bar chart, two rows of squares indicate the statistical significance of comparisons based on karyotype (2La *versus* 2L+^a^; “Inversion”) and environmental condition (arid *versus* benign; “Condition”). Squares are color coded according to level of significance; those with white circles represent *P*-values that remain significant following Bonferroni adjustment for multiple testing.

Epicuticular hydrocarbon composition was examined in *An. coluzzii* (SUCAM), using eight day-old virgin females of both (2La or 2L+^a^) karyotypes, maintained under benign or arid conditions. Regardless of karyotype or environmental condition, the same 16 peaks were consistently observed in all chromatograms, suggesting that these two factors had no qualitative effect on CHC composition (Figure 5). Hydrocarbon identification, based on mass spectra and retention times relative to known standards, revealed several alkanes, alkenes, and methyl-branched alkanes with base chain lengths ranging from 23 to 33 carbons; one compound (peak 14; KI 3158) could not be identified (Table 3). Peaks 4, 7, and 13 (heptacosane, nonacosane, and 13-methylhentriacontane, respectively) were the most abundant, accounting for about 61 to 68% of the total CHC profile (Figures 5 and 6).

**Table 3:** **Identity of the 16 epicuticular hydrocarbons detected from** ***An. coluzzii*** **SUCAM**

**Peak number**	**Peak identity**	**Abbreviation**	**Retention index**
1	Tricosane	n-C_23_	2299
2	Pentacosane	n-C_25_	2499
3	Hexacosane	n-C_26_	2599
4	Heptacosane	n-C_27_	2700
5	Octacosane	n-C_28_	2800
6	1-Nonacosene	1-C_29_	2879
7	Nonacosane	C_29_	2900
8	7-; 9-; 11- 13-; 15-Methylnonacosane	7-; 9-; 11- 13-; 15-C_29_	2932
9	Triacontane	C_30_	3000
10	11-;12-;13-;14-;15-Methyltriacontane	11-;12-;13-;14-;15-C_30_	3031
11	1-Hentriacontene	1-C_31_	3081
12	Hentriacontane	C_31_	3101
13	13-Methylhentriacontane	13-C_31_	3129
14	unknown	unknown	3158
15	Tritriacontane	C_33_	3300
16	9-;11-;13-;15-;17-Methyltritriacontane	9-;11-;13-;15-;17-C_33_	3334

Assessment of quantitative differences in the 16 CHCs between treatment groups was conducted two ways, by compound and across the CHC profile. Compound-by-compound ANOVA tests revealed six CHCs whose relative abundance was significantly different owing either to karyotype or environmental condition, and an additional four CHCs were responsive to both factors (Figure 6), but no significant interaction was detected between karyotype and environment. Of the seven compounds responsive to karyotype (peaks 1, 5, 6, 7, 9, 10, 11), all save one (7, nonacosane) were more abundant in the 2La background. Interestingly, the relative proportion of nonacosane was significantly lower, while the relative proportions of two alkenes (peak 6, 1-nonacosene; and peak 11, 1-hentriacontene) and one methyl-branched alkane (peak 10, methyltriacontane) were significantly higher in the 2La background. Lower amounts of an unbranched alkane in favor of more alkenes and a branched alkane should disrupt lipid packing and lower the melting point relative to 2L+^a^[36]. Seven compounds were responsive to environmental condition. Of those, five were unbranched alkanes that increased in proportion in arid-acclimated females (peaks 5, 7, 9, 12, 15). Two compounds decreased in response to aridity, an alkene (peak 6, 1-nonacosene) and the unknown (peak 14). Given that double bonds and other chain modifications disrupt lipid packing, and that the unknown compound decreased under arid conditions, it is tempting to speculate that this compound may be an alkene or a branched alkane.

**Figure 7:**
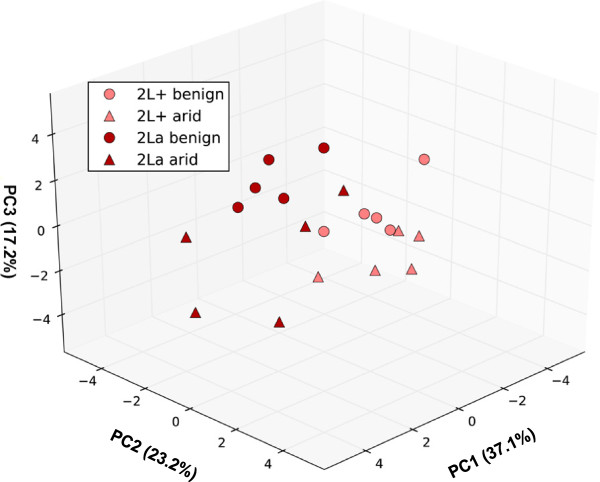
**Principal component analysis of CHC profiles for four karyotype and environment treatment groups (20 samples).** Shown are the first three PCs. Each symbol represents one sample, coded by color and shape to indicate karyotype (light red, 2L+^a^; dark red, 2La) and environment (benign, circle; arid, triangle). The percentage of variance represented by each PC is indicated on the axis.

**Figure 8:**
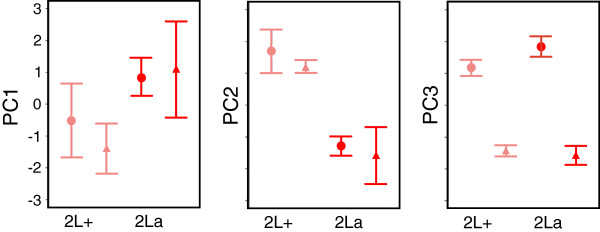
**Mean PC score (±SE) for the first three PCs by karyotype and environmental condition.** Color and shape indicate karyotype (light red, 2L+; dark red, 2La) and environment (benign, circle; arid, triangle).

Principal component analysis (PCA) was used to compare overall CHC profiles. The first three PCs accounted for 77.5% of the total variance (Figure 7, Table 4). The factor loadings (the correlation between individual compounds and the PC) above 0.25 were used to identify the compounds most influential for each of the first three PCs (Table 4), to guide interpretation of the biological significance of each vector [47]. PC1 was weighted negatively by peaks 2, 4 and 7 (pentacosane, heptacosane, nonacosane), and positively by peaks 10, 11, 13, and 16 (three methyl-branched alkanes and an alkene). Thus, PC1 may describe trade-offs between shorter chain alkanes that were relatively more abundant in the 2L+^a^ background—especially under arid conditions—and longer CHCs with chain modifications that were more abundant in the 2La background, especially under benign conditions (Figure 6). That PC1 describes a karyotype effect, particularly for 2L+^a^ under arid conditions and 2La under benign conditions, is reflected in a plot of the mean PC1 scores by karyotype and environmental condition (Figure 8A). PC2 was negatively weighted by peaks 1, 3, 5, 6, and 8–10, including an alkene and two methyl-branched alkanes; all of the corresponding compounds were more abundant in 2La karyotypes relative to their 2L+^a^ counterparts (Figure 6). Accordingly, PC2 seems to be a vector that describes variation in CHC profiles in response to karyotype differences, as suggested by Figure 8B. PC3 was negatively weighted by peaks 7, 9, 12, and 15, corresponding to the longer unbranched alkanes that were proportionally more abundant in arid-acclimated females. PC3 also was negatively weighted by peak 14, the unknown compound that decreased in abundance in response to aridity (Figure 6). Therefore, PC3 is a vector that explains variation in CHC profiles due to environmental conditions (Figure 8C). Multivariate analysis using the PCs with eigenvalues greater than 1 as response variables, and karyotype, environment and their interaction as fixed factors, indicated that karyotype and environment, but not their interaction, were significant influences (Table 5).

**Table 4:** Factor loadings for each compound based on results of principal component analysis of CHC profiles

**Compound (abbreviation)**	**PC1 (37.1%)**	**PC2 (23.2%)**	**PC3 (17.2%)**
1 (n-C_23_)	-0.037	**-0.409**	0.059
2 (n-C_25_)	**-0.351**	-0.145	0.006
3 (n-C_26_)	-0.202	**-0.382**	0.043
4 (n-C_27_)	**-0.364**	-0.028	0.205
5 (n-C_28_)	-0.034	**-0.409**	**-0.246**
6 (1-C_29_)	0.186	**-0.324**	0.218
7 (C_29_)	**-0.260**	0.216	**-0.334**
8 (7-; 9-; 11- 13-; 15-C_29_)	0.106	**-0.257**	0.056
9 (C_30_)	0.180	**-0.283**	**-0.333**
10 (11-;12-;13-;14-;15-C_30_)	**0.323**	**-0.251**	-0.002
11 (1-C_31_)	**0.338**	-0.095	0.205
12 (C_31_)	0.161	0.196	**-0.467**
13 (13-C_31_)	**0.320**	0.197	0.214
14 (unknown)	0.185	0.219	**0.412**
15 (C_33_)	0.224	0.041	**-0.347**
16 (9-;11-;13-;15-;17-C_33_)	**0.352**	-0.011	-0.154

Bold, loading factors >0.25.

**Table 5:** Multivariate analysis of variance (MANOVA) testing the effect of karyotype, environmental condition and their interaction on the overall CHC profile

	**Pillai’s trace**	***F***	**d.f.**	***P***
Karyotype	0.786	11.968	4,13	<0.001
Environment	0.906	31.517	4,13	<0.001
Karyotype x environment	0.197	0.796	4,13	0.549

## Discussion

Climate is a major factor limiting the geographic and seasonal ranges of insects [48]. Climatic variables, particularly those related to water availability, are effective predictors of the distribution of African malaria vectors in the *Anopheles gambiae* complex and the chromosomal inversion polymorphisms that segregate within them [49-52]. Physiological studies of *An. gambiae* and *An. coluzzii*, based on field isolates as well as laboratory colonies, suggest that the superior desiccation resistance afforded by alleles linked to inversion 2La and other inversions on chromosome 2 are major determinants responsible for their relative abundance under arid conditions [15,17,53]. Both species can mount a plastic response to desiccation stress, as acclimation to arid conditions increases their resistance [17]. However, the differential desiccation resistance of alternative 2La karyotypes in an otherwise common genetic background, under controlled and identical environmental conditions, indicates that physiological differences between karyotypes have a genetic basis [17]. Genomic scans of elevated sequence divergence between karyotypes suggested a possible role for cuticle protein genes [23,28]. This study aimed to test whether alternative 2La karyotypes were associated with differences in cuticle characteristics that could contribute to differential desiccation resistance.

Mean cuticle thickness, when standardized for body weight, differed between alternative karyotypes, although the difference was statistically significant only in *An. coluzzii* maintained under benign conditions. Moreover, in both species, environmental condition—whether benign or arid—did not alter the relationship between cuticle thickness and karyotype. Recently, Vannini *et al.*[40] studied the location of the structural cuticular protein CPF3, whose transcripts are significantly more abundant in virgin females of *An. coluzzii* relative to *An. gambiae*[54]. The location of this protein in appendage exocuticle was deemed inconsistent with a role for CPF3 in pheromone display for mate recognition, as proposed previously [54,55]. However, Vannini *et al.*[40] suggested that its exocuticular location and higher transcript abundance in *An. coluzzii* might be related to the greater desiccation resistance of this species [53]. If so, CPF3 may contribute either to a more densely packed or a thicker epicuticular layer in *An. coluzzii*. The CPF3 gene is not located inside the 2La inversion, and there is no *a priori* expectation that CPF3 should be differentially expressed between 2La karyotypes of *An. coluzzii*, but comparisons between species (by karyotype and condition) suggest that if anything, *An. coluzzii* has the thinner cuticle of the two species.

Within *An. coluzzii*, mean cuticle thickness was greater for 2La than 2L+^a^ females at eight days post-eclosion. If a thicker cuticle were a better barrier to water loss, the expectation would be that females with thicker cuticles should lose water at a lower rate. However, physiological studies showed the contrary; based on the same *An. coluzzii* SUCAM colonies, it was found that eight day old *An. coluzzii* 2La females actually lost water at a significantly higher rate than their 2L+^a^ counterparts with thinner cuticles [17]. Taken together, these data suggest that for these anophelines, increased cuticle thickness *per se* does not appear to reduce water loss and is unlikely to be an adaptation to desiccation stress, although this trait has been implicated in resistance of *Anopheles funestus* to insecticides [37]. A caveat to this conclusion is that cuticle thickness was only measured from an appendage; we cannot rule out the possibility that cuticle thickness elsewhere in the body increased in response to aridity.

Cuticular hydrocarbons are the dominant constituents of the waxy epicuticular layer that coats the insect cuticle, thought to be the main barrier to water loss. We were able to detect 16 CHCs in *An. coluzzii* females in common with previous studies of *An. gambiae* and *An. coluzzii* field populations [56,57], but were unable to detect the dimethyl-branched CHCs (chain length C_33_-C_45_) observed in those studies despite similar methods. Based on the 16 CHCs detected, we found no qualitative differences in CHC composition between karyotypes or environmental conditions. However, we did find quantitative differences that were consistent with expectations. Exposure to arid conditions caused a decrease in the relative proportions of unsaturated CHCs and those with methyl-branched chains in favor of unbranched, saturated CHCs in both karyotypes, an effect expected to enable denser packing of the lipid layer, hence a higher melting point and superior waterproofing [36]. In terms of karyotype-associated CHC profiles, the 2L+^a^ karyotype was characterized by a greater proportion of saturated and unbranched hydrocarbon chains relative to the 2La karyotype, apparently lending 2L+^a^ females better waterproofing under desiccation stress. Although this result seems counter-intuitive given the association of 2La (and not 2L+^a^) with aridity in natural populations, it is entirely consistent with previous physiological studies demonstrating that *An. coluzzii* 2La females lost water at a higher rate than their 2L+^a^ counterparts, at eight days post-eclosion [17]. Those same physiological studies found that at the vulnerable teneral stage when the cuticle is still hardening, 2La females lost water at a significantly *lower* rate than 2L+^a^ females, potentially explaining the advantage of the 2La karyotype (more precisely, the advantage of causal alleles linked to 2La) in arid environments. In fact, the water loss rate for 2La females was essentially constant from adult eclosion to eight days post-eclosion; the difference in water loss rates between karyotypes was a function of the reduction of the rate of water loss in 2L+^a^ females during that time-frame [17].

The challenge for insects in desiccating environments is to maintain their water balance. One important component of the overall strategy of water regulation, considered in this study, is to conserve water by minimizing its loss via the route responsible for the greatest body water loss, the cuticle [34]. However, there are other components of insect water economy that were not considered here, including physiological adjustments following a blood meal [35], that operate in concert to determine the ultimate desiccation resistance phenotype. During the off-host stage (*sensu*[35]), mosquitoes may increase their water intake by imbibing more nectar or other plant juices. Additionally, they may limit water loss by resting in humid microenvironments, and avoiding activity during the relatively hot, dry daytime hours. Water loss may also be limited by increasing body size, thereby reducing the surface-to-volume ratio [58]—a strategy that both *An. gambiae* and *An. coluzzii* seem to have adopted in our experiments based on their increased dry mass under arid conditions (Figure 2). Increasing tolerance to desiccation is another non-exclusive strategy, a process that may be aided by heat shock proteins such as Hsp70 and Hsp90 [59,60]. In this regard, it is intriguing that a tandem trio of Hsp90 genes are found inside the 2La inversion, in a region of exceptionally high sequence divergence from 2L+^a^ that has been implicated in the selective maintenance of the inversion [28]. Not least, desiccation resistance may be improved by boosting glycogen content, as this molecule may be used to store water [30]. Indeed, this may be one of the primary ways in which alleles within the 2La inversion contribute to desiccation resistance, as Gray *et al*. [17] found that mass-specific body water content was significantly higher at four and eight days post-eclosion in 2La versus 2L+^a^ females, a circumstance that presumably compensated for the significantly higher rate of water loss observed in eight day-old 2La females.

## Conclusions

There remains much to be learned of the underlying molecular mechanisms governing the complex and sophisticated phenotype that is desiccation resistance. Yet ability to withstand desiccation is a primary determinant of the geographic, microgeographic, and seasonal distribution of malaria vectors. The difficulty of detecting even the most desiccation resistant *An. coluzzii* mosquitoes in the West African Sahel strongly testifies to the limits that aridity poses to these vectors [61,62]. The differential effects of alternative inversion karyotypes on mosquito behaviors such as endophily, which can be interpreted in terms of optimal habitat choice with respect to degree of aridity, also have important impacts on the likelihood of vector-human contact and vector contact with indoor-based vector control [63]. Thus, they affect vectorial capacity and the epidemiology of malaria. Importantly, although the 2La inversion is associated with superior desiccation resistance and its frequencies peak in *An. gambiae* and *An. coluzzii* populations living in arid climates, the same inversion is rare or absent in populations from mesic environments, implying fitness tradeoffs. Deeper understanding of water regulation and its limits should uncover the hidden costs as well as the benefits of water balance strategies, information that could be exploited for vector control.

## Competing interests

The authors declare no competing interests.

## Authors’ contributions

KR, NB and ZS conceived the study. CC selected the NDKO subcolonies of known karyotype. KR performed mosquito husbandry and treatments, and subsequent measurements of total dry body mass, cuticle thickness by SEM, and CHC composition by gas chromatography. KR, FL, CL and ZS analyzed results. KR, NB and ZS wrote the manuscript. All authors read and approved the final manuscript.
